# Prognostic significance of abdominal aortic calcification scores on dual-energy X-ray absorptiometry scans for mortality in cancer survivors: NHANES-based cohort study (2013–2019)

**DOI:** 10.1093/ehjopen/oeaf116

**Published:** 2025-09-01

**Authors:** Mustafa Al-jarshawi, Glen P Martin, Andrew Cole, Miguel Nobre Menezes, Richard K Cheng, Juan Lopez-Mattei, Eric H Yang, Mamas A Mamas

**Affiliations:** Keele Cardiovascular Research Group, Centre for Prognosis Research, Keele University, Stoke-on-Trent, UK; Centre for Health Informatics, Division of Informatics, Imaging and Data Science, Faculty of Biology, Medicine and Health, University of Manchester, Manchester M13 9PL, UK; Centre for Health Informatics, Division of Informatics, Imaging and Data Science, Faculty of Biology, Medicine and Health, University of Manchester, Manchester M13 9PL, UK; Keele Cardiovascular Research Group, Centre for Prognosis Research, Keele University, Stoke-on-Trent, UK; Structural and Coronary Heart Disease Unit, Cardio-Oncology Unit CHULN Hospital de Santa Maria, Cardiovascular Center of the University of Lisbon, 1649-028 Lisbon, Portugal; Division of Cardiology, University of Washington, Seattle, WA 98195-6422, USA; Division of Cardiology, Lee Health Heart Institute, Fort Myers, FL 33908, USA; UCLA Cardio-Oncology Program, Division of Cardiology, Department of Medicine, University of California at Los Angeles, Los Angeles, CA 90095, USA; Keele Cardiovascular Research Group, Centre for Prognosis Research, Keele University, Stoke-on-Trent, UK

**Keywords:** Abdominal aortic calcification (AAC), Cancer survivors, Cardiovascular mortality, All-cause mortality, Dual-energy X-ray absorptiometry (DXA), Cardio-oncology

## Abstract

**Aims:**

Abdominal aortic calcification (AAC) is a marker of systemic atherosclerosis associated with adverse cardiovascular (CV) outcomes in the general population. This study aimed to evaluate the association of AAC with all-cause and CV mortality in cancer survivors.

**Methods and results:**

Using 7 years of data from the National Health and Nutrition Examination Survey (NHANES, 2013–2019), we analysed a nationally representative cohort of US cancer survivors. AAC burden was quantified using the Kauppila AAC-24 scores on dual-energy X-ray absorptiometry (DXA) scans. Kaplan–Meier curves and multivariable Cox models were used to assess the associations between AAC and all-cause mortality, while Fine and Gray models assessed associations between AAC and CV mortality, accounting for non-CV mortality as a competing risk. A total of 23 126 424 cancer survivors (aged ≥40 years) were analysed, recording 4 199 131 (114 unweighted) all-cause deaths and 1 160 618 (34 unweighted) CV deaths over a 69-month median follow-up. AAC was present in 46%, with 19.5% of the cohort showing severe AAC (AAC-24 > 6). Each one-unit increase in AAC-24 score was associated with higher risks of all-cause mortality and CV mortality [adjusted hazard ratio, 95% confidence interval of 1.04 (1.00–1.09) and subdistribution hazard ratio 1.07 (1.02–1.12); *P* = 0.047 and *P* = 0.002, respectively] after adjustment for demographic, socioeconomic, traditional CV risk factors, baseline comorbidities, and cancer-specific characteristics.

**Conclusion:**

AAC detected on DXA scans is independently associated with increased all-cause and CV mortality in cancer survivors aged 40 years and older. DXA-based AAC assessment may serve as a valuable tool for risk stratification in cardio-oncology.

## Graphical Abstract

**Graphical Abstract:**
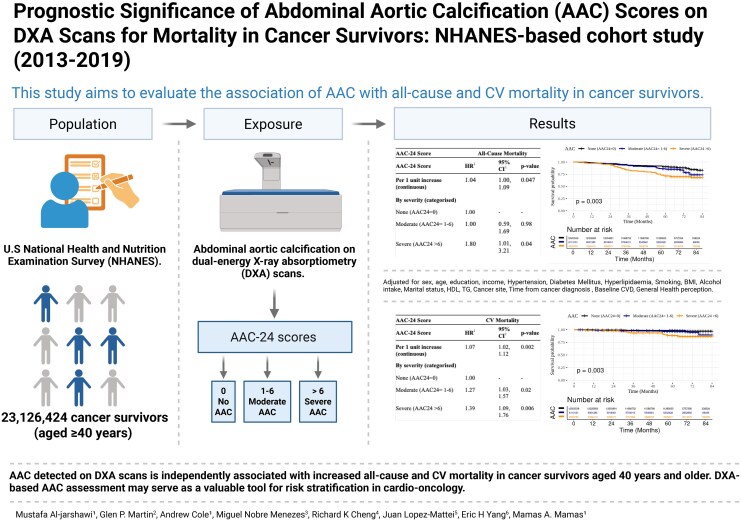

## Introduction

Atherosclerosis is a chronic, systemic, and progressive vascular disease characterized by the accumulation of lipid-rich plaques in the arterial walls.^1^ Over time, these plaques undergo calcification, leading to arterial stiffness, reduced vascular compliance, and impaired blood flow regulation. While atherosclerosis predominantly affects the coronary and carotid arteries, it is also prominent in the aorta, where extensive calcification has been linked to an increased risk of cardiovascular (CV) morbidity and mortality.^2^ Abdominal aortic calcification (AAC) represents an early site of both intimal and medial vascular calcification and is associated with multisite atherosclerosis and cardiovascular disease (CVD) risk.^3,4^

Studies have demonstrated that AAC is strongly correlated with calcification in other arterial beds, including the coronary and carotid arteries, even in adults without manifest CVD.^5,6^ A growing body of epidemiological evidence suggests that AAC is a strong predictor of adverse CV events and mortality, independent of traditional CV risk factors.^7–9^ Longitudinal studies have also demonstrated that individuals with higher AAC scores have an increased risk of myocardial infarction, stroke, and coronary heart disease.^10–13^

Additionally, AAC has demonstrated a stronger association with all-cause mortality compared to coronary artery calcification (CAC), suggesting that it may offer better prognostic value.^8,14^ Unlike CAC, AAC involves both the intimal and medial layers of the arterial wall, which may contribute to this stronger prognostic significance.^3^ However, despite these associations, AAC’s prognostic value beyond CAC remains underexplored in the cancer population, where systemic atherosclerosis may play a crucial role in long-term health outcomes. Most studies exploring the prognostic impact of AAC have focused on the general population, older adults, or individuals with chronic kidney disease,^2,15,16^ leaving gaps in understanding its relevance among other populations, such as cancer survivorship.

Cancer survivors represent a unique, high-risk group, as they frequently experience accelerated vascular ageing and atherosclerosis due to cancer and therapy-related factors, which may exacerbate vascular calcification and CVD risk.^17^ However, the prevalence, severity, and prognostic significance of AAC in cancer survivors remain largely unknown beyond its role in predicting postoperative surgical outcomes, which have been assessed only in small cohorts with a limited range of cancer types.^18–22^ This study aims to address this gap by evaluating the association between AAC score, as a marker of systemic atherosclerosis, and long-term clinical outcomes (all-cause and CV mortality) across multiple cancer types, using a nationally representative cohort.

## Methods

### Data sources

This analysis used data from the National Health and Nutrition Examination Survey (NHANES), which we restricted to adult participants (aged ≥40 years) with a diagnosis of cancer as the NHANES does not provide AAC scores for participants aged <40 years. The National Center for Health Statistics, part of the Centers for Disease Control and Prevention (CDC), produces this survey aimed at monitoring the health of the US population. The NHANES is a major programme that generates a nationally representative sample of the civilian non-institutionalized US population from ∼5000 individuals each year in a 2-year cycle. These are generated through complex, multistage, probability sampling process with oversampling of specific subgroups to improve the reliability and accuracy in these populations.^23^ Detailed sampling and data collection procedures have been previously published.^24^ In addition to demographics, socioeconomic, and health-related questions, the surveys also include medical, physiological, and laboratory measurements.

### Study design and population

This study was a retrospective cohort study that used data from the 2013–2014 NHANES cycles. Data were linked with the NHANES Linked Mortality File, which links participants of the NHANES aged 18 and above with death records in the National Death Index dataset through 31 December 2019, the latest mortality data available.^25^

### Study sample

Survey responders who answered ‘yes’ to the question, ‘Have you ever been told by a doctor or other health professional that you had cancer or a malignancy of any kind?’ in the medical conditions questionnaire were identified as patients with cancer and included in this analysis. Those identified were further asked, ‘What kind of cancer?’, and responses were recorded to specify the malignancy organ/type. The cancer organs/types considered in our analysis included haematological (lymphoma and leukaemia), bone, brain, breast, genitourinary (kidney, bladder, and prostate), gynaecological (ovarian and uterine), gastrointestinal (oesophageal, stomach, and colon), liver, gallbladder, pancreas, skin (melanoma, non-melanoma, and others), soft tissue, and head and neck (thyroid, larynx, mouth, tongue, and lip) cancers. Participants were excluded if they were ineligible for mortality follow-up or had incomplete data for the exposure variable or covariates. The study flowchart is provided in Supplementary material online, *Figure S1*.

### Exposure

AAC was the primary exposure variable in this study. AAC was assessed using lateral dual-energy X-ray absorptiometry (DXA) scans of the thoracolumbar spine, conducted as part of the 2013–2014 NHANES mobile examination centre (MEC) assessments. The DXA examinations were administered by trained and certified radiology technologists. Several studies have found that lateral spine images obtained with DXA for vertebral fracture assessment (VFA) can also detect AAC with reasonably good sensitivity and specificity.^11,26^ The image resolution of lateral spine scans obtained with DXA is close to the image resolution of a standard radiograph, but the DXA scan produces much lower radiation exposure. Scans were performed on eligible survey participants who were 40 years of age and older, using Hologic Discovery Model A densitometers (Hologic, Marlborough, MA, USA) with Apex version 3.2 software. The detailed data collection procedures are available on the CDC NHANES website (https://wwwn.cdc.gov/Nchs/Nhanes/2013-2014/DXXAAC_H.htm#DXDAACST). Participants were excluded from DXA examination if they were under 40 years of age, pregnant, had undergone radiographic contrast material (barium) exposure within the past 7 days, weighed >450 pounds, or had a Harrington rod in the spine for scoliosis.

### Abdominal aortic calcification score quantification

AAC severity was quantified using the Kauppila scoring system, a well-established semi-quantitative method.^27^ The anterior and posterior aortic walls were divided into four segments, corresponding to the lumbar vertebrae L1–L4. Each segment was visually evaluated for aortic calcification, and a score between 0 and 3 was assigned based on the extent of calcification:

- 0 = no calcification
- 1 = calcification of ≤1/3 of the aortic wall in that segment
- 2 = calcification of >1/3 but <2/3 of the aortic wall
- 3 = calcification of ≥2/3 of the aortic wall

Scores for the anterior and posterior walls were assessed separately, resulting in a total AAC score ranging from 0 to 24.

### Baseline characteristics and covariates

Baseline demographic, socioeconomic, lifestyle, clinical, and laboratory characteristics were collected through standardized questionnaires and objective measurements in MEC assessments at the same time as AAC measurement. Demographic characteristics included age, sex, and race/ethnicity. Age was recorded as a continuous variable. Sex was classified as male or female, while race and ethnicity were self-reported and categorized as Mexican American, other Hispanic, non-Hispanic White, non-Hispanic Black, non-Hispanic Asian, and other race (including multiracial individuals). Socioeconomic factors included educational attainment, income, and marital status. Education level was categorized as less than high school, high school or equivalent, or more than high school. The ratio of family income to poverty level (PIR) was used to assess socioeconomic status and was stratified into <1.31, 1.31–1.85, and 1.86–3.5. Marital status was classified as married, widowed, divorced, separated, never married, or living with a partner. Lifestyle factors included smoking status, alcohol consumption, and general health perception. Smoking status was categorized as never, former, or current smoker, while alcohol intake was classified as none, moderate, or heavy. Self-reported general health status was categorized as excellent, very good, good, fair, or poor.

Anthropometric and clinical measurements included body mass index (BMI) and blood pressure. BMI was calculated as weight in kilograms divided by height in metres squared (kg/m²). Blood pressure was measured in accordance with NHANES standardized protocols, with three consecutive readings recorded and the mean value used for analysis. Both systolic and diastolic blood pressure were included as continuous variables.

Laboratory parameters were obtained from venous blood samples, processed according to NHANES laboratory protocols. The lipid profile included total cholesterol (mmol/L), direct HDL cholesterol (mmol/L), LDL cholesterol (mmol/L), and triglycerides (TG) (mmol/L). Glycohaemoglobin (HbA1c, %) was measured as an indicator of blood glucose control. Renal function was assessed using serum creatinine (µmol/L) and estimated glomerular filtration rate (eGFR, mL/min/1.73 m²), calculated using the Modification of Diet in Renal Disease (MDRD) formula. Additional biochemical markers included serum albumin (g/dL) and total calcium (mmol/L). Adjusted calcium levels were derived from total calcium measurements and corrected for serum albumin levels using the standard formula: Adjusted Calcium (mmol/L) = Total Calcium (mmol/L) + 0.02 × [40 − Albumin (g/L)].

Cardiovascular risk factors and comorbidities were also considered. Hypertension, hyperlipidaemia, and diabetes mellitus were defined based on self-reported previous medical records from a healthcare professional or physician. Baseline CVD was determined by participants’ affirmative responses to a diagnosis of congestive heart failure, coronary heart disease, angina/angina pectoris, heart attack, or stroke. Cancer-related variables included time from cancer diagnosis (in years) and cancer site, both of which were recorded and included in this analysis. Time from cancer diagnosis was calculated as the difference between the participant’s self-reported age at the time of NHANES assessment and their self-reported age at the first cancer diagnosis.

Covariates included in the outcomes analysis were demographic variables (age, sex, and race/ethnicity), socioeconomic variables (marital status, education, and income), behavioural factors (alcohol status, general health perception), body parameters (BMI), laboratory parameters (direct HDL cholesterol and trigylcerides), traditional CV risk factors (diabetes, hypertension, smoking history, and hyperlipidaemia), comorbid CVD, cancer type, and time from cancer diagnosis. These covariates were selected based on clinical relevance and prior literature.

### Outcomes

The primary outcome of interest in this study was all-cause mortality, with a secondary outcome of CV mortality (International Statistical Classification of Diseases, 10th revision codes I00–I09, I11, I13, I20-I51, and I60–I69). Mortality status was assessed via a probabilistic record match to death certificate records from the National Death Index. Additional sources were used to determine mortality status, including those obtained via linkages with the US Social Security Administration and/or by active follow-up of survey participants. Follow-up time for each outcome was counted in months from the baseline examination date until the registered date of death or the end of the study (31 December 2019), whichever occurred first.

### Ethical approval

The NHANES dataset research protocol was approved by the National Center for Health Statistics Ethics Review Board, and all participants signed informed consent. Since the data are publicly available, individual consent for this analysis was not required, and this research was exempt from institutional review board approval. This study adhered to the ethical principles for medical research as outlined in the Declaration of Helsinki.

### Statistical analysis

All statistical analyses were based on weighted records and conducted using R (R Foundation for Statistical Computing, Vienna, Austria) and SPSS version 28.0.0 (IBM, Armonk, NY, USA). NHANES’ MEC weights were applied to account for oversampling, non-response, and non-coverage, ensuring nationally representative estimates. Combined weights for the survey cycles 2013–2014 were calculated and applied following the NHANES-recommended formulae for combining weights across the survey cycles. The data weighting variable was subsequently rounded to the nearest integer. A detailed explanation of the weighting methods is available on the NHANES website (https://wwwn.cdc.gov/nchs/nhanes/tutorials/weighting.aspx).

The weighted sample size adequacy for prognostic modelling in this study was assessed based on the criteria proposed by Riley *et al*. (2020)^28^ using the pmsampsize package in R,^29^ which determines the minimum required sample size for time-to-event outcomes in prognosis studies while considering the number of candidate predictor parameters, event rate, and anticipated model performance to ensure sufficient power and minimize the risk of model overfitting. Assumed inputs to the calculations, and the outputted minimum required weighted sample size are detailed in (see Supplementary material online, *Table S1*).

The normality of data distribution was assessed using the Shapiro–Wilk test. Continuous variables are reported as medians with interquartile ranges (IQR), while categorical variables are expressed as proportions. Participants were classified based on AAC burden, with severe AAC defined as an AAC-24 score >6, in line with previous studies.^16,30,31^ Moderate AAC was defined as an AAC-24 score between 1 and 6, while no AAC was assigned to participants with an AAC-24 score of 0. Comparisons between variables were conducted using the design-based Kruskal–Wallis test for continuous variables and Pearson’s *χ*² test with the Rao and Scott adjustment for categorical variables to account for the complex survey design. Both univariable- and multivariable-adjusted analyses were conducted. Survival distributions between groups were assessed using the log-rank (Mantel–Cox) test and plotted in Kaplan–Meier survival curves.

In the main analysis, Cox proportional hazards (PH) regression models were fitted to evaluate the relationship between AAC-24 scores and all-cause mortality outcome. For CV mortality, a competing risk analysis was conducted using Fine and Gray semiparametric proportional hazards models, which accounted for the competing risks of non-CV mortality and were used to calculate subdistribution hazard ratios (sHRs).^32,33^ Four models were developed in each analysis to account for potential confounders. Covariates for each model were selected *a priori* based on established cardiovascular risk factors, prior literature, and their clinical relevance in cancer survivorship. No data-driven variable selection methods were used. Model 1 was unadjusted, assessing the crude association between AAC-24 and mortality outcomes. Model 2 adjusted for key demographic, socioeconomic, and traditional CV risk factors (age, sex, education, income, hypertension, diabetes mellitus, hyperlipidaemia, smoking status, and BMI). Model 3 further adjusted for other key variables reflecting lifestyle factors, cancer site, comorbidities, and self-reported health status [alcohol intake, marital status, TG, direct HDL cholesterol, cancer site, time from cancer diagnosis, baseline CVD, and general health perception] to account for additional confounders. Model 4 included the same covariates as Model 3 but analysed AAC as a categorical variable to enhance clinical interpretability based on severity groups, with no AAC (AAC-24 = 0) as the reference group. In addition, we refitted the fully adjusted models (Model 3 for continuous AAC-24 scores and Model 4 for AAC-24 scores categories by severity group) in a supplementary analysis restricted to cancer types with representation across all three AAC strata, to further assess the robustness of associations and reduce potential confounding by cancer type. Follow-up time was measured from the date of the NHANES examination until death or the end of the study (31 December 2019) and was used as the time-to-event variable. Interaction terms with the primary exposure variable (AAC) were examined to check for any potential effect modification by age, sex, smoking status, CVD status, diabetes, and hypertension. However, no statistically significant interactions were observed (*P* > 0.05).

The PH assumption for the Cox models was evaluated using Schoenfeld residual testing, which indicated no significant global correlation between model residuals and time, confirming that the PH assumption was not violated (see Supplementary material online, *Table S2*). To assess potential multicollinearity, variance inflation factor (VIF) analysis was conducted, with no variables exceeding the exclusion threshold (VIF ≥ 5 to 10) (see Supplementary material online, *Table S3*), consistent with established guidelines in the literature.^34^ The predictive performance of the fitted models was assessed using calibration slope and Harrell’s C-statistic measures, with adjustment for in-sample optimism performed through internal validation via bootstrapping (with 1000 samples). Participants with pre-existing CVD and hypercalcaemia (defined by adjusted calcium >2.6 mmol/L) were excluded from the sensitivity analysis. The impact of AAC-24 scores in this sub-cohort on long-term (7-year) all-cause and CV mortality was reassessed using the same statistical analysis.

## Results

A total of 23 126 424 weighted records were included in the analysis, representing individuals after applying survey weights. All participants were adults (≥40 years old), with complete vital status data.

### Baseline characteristics

The median age of the cohort was 66 years (IQR: 57–75), with 46% being male. The racial distribution was predominantly non-Hispanic White (88%), followed by non-Hispanic Black (4.9%) and Mexican American (2.5%). CV risk factors were prevalent, with 60% of participants having hypertension, 17% diagnosed with diabetes mellitus, and 39% classified as current smokers. CV comorbidities were also common, with 19% of participants having a history of baseline CVD, 7% reporting a history of congestive heart failure, 6.8% with coronary heart disease, 6.1% with a previous heart attack, 2.5% reporting angina, and 6.5% having a history of stroke (*Table 1*).

**Table 1:** Survey-weighted baseline characteristics

	AAC				
Variable	Overall (AAC24 = 0–24) *n* = 23 126 424^a^	None (AAC24 = 0) *n* = 12 503 539^a^	Moderate (AAC24 = 1–6) *n* = 6 113 131^a^	Severe (AAC24 > 6) *n* = 4 509 754^a^	*P*-value^b^
Unweighted records	490 (100%)	237 (48.36%)	151 (30.81%)	102 (20.81%)	
All-cause mortality	4 199 131 (18%)	1 596 115 (13%)	1 216 905 (20%)	1 386 110 (31%)	0.007
CV mortality	1 160 618 (5.0%)	300 350 (2.4%)	337 428 (5.5%)	522 840 (12%)	0.011
Age in years	66 (57, 75)	62 (56, 70)	69 (58, 77)	74 (67, 80)	<0.001
Age group					0.003
40–60	7 331 596 (32%)	5 098 285 (41%)	1 698 773 (28%)	534 538 (12%)	
>60	15 794 829 (68%)	7 405 254 (59%)	4 414 358 (72%)	3 975 216 (88%)	
Sex					0.3
Male	10 671 676 (46%)	6 025 093 (48%)	2 354 522 (39%)	2 292 062 (51%)	
Female	12 454 749 (54%)	6 478 447 (52%)	3 758 610 (61%)	2 217 693 (49%)	
Race/Hispanic origin					0.8
Mexican American	570 117 (2.5%)	314 253 (2.5%)	168 335 (2.8%)	87 529 (1.9%)	
Other Hispanic	427 517 (1.8%)	257 879 (2.1%)	154 416 (2.5%)	15 222 (0.3%)	
Non-Hispanic White	20 283 631 (88%)	10 913 274 (87%)	5 308 236 (87%)	4 062 121 (90%)	
Non-Hispanic Black	1 128 444 (4.9%)	623 755 (5.0%)	331 155 (5.4%)	173 533 (3.8%)	
Non-Hispanic Asian	333 512 (1.4%)	190 756 (1.5%)	71 271 (1.2%)	71 485 (1.6%)	
Other race, including multiracial	383 205 (1.7%)	203 622 (1.6%)	79 719 (1.3%)	99 864 (2.2%)	
Education level					0.066
Less than high school	647 283 (2.8%)	232 273 (1.9%)	183 210 (3.0%)	231 800 (5.1%)	
High school or equivalent	5 495 851 (24%)	2 581 895 (21%)	1 890 488 (31%)	1 023 467 (23%)	
More than high school	16 983 292 (73%)	9 689 371 (77%)	4 039 433 (66%)	3 254 487 (72%)	
Ratio of family income to poverty					0.3
<1.31	3 465 322 (15%)	1 755 714 (14%)	1 099 349 (18%)	610 259 (14%)	
1.31–1.85	2 354 803 (10%)	892 408 (7.1%)	784 521 (13%)	677 874 (15%)	
1.86–3.5	17 306 300 (75%)	9 855 417 (79%)	4 229 262 (69%)	3 221 622 (71%)	
Marital status					0.3
Married	14 501 206 (63%)	8 142 237 (65%)	3 909 069 (64%)	2 449 900 (54%)	
Widowed	3 314 629 (14%)	1 306 248 (10%)	927 079 (15%)	1 081 302 (24%)	
Divorced	3 022 900 (13%)	1 672 609 (13%)	707 907 (12%)	642 384 (14%)	
Separated	311 481 (1.3%)	145 775 (1.2%)	145 459 (2.4%)	20 248 (0.4%)	
Never married	1 501 708 (6.5%)	922 539 (7.4%)	312 153 (5.1%)	267 016 (5.9%)	
Living with partner	446 673 (1.9%)	314 132 (2.5%)	83 637 (1.4%)	48 904 (1.1%)	
Body mass index (kg/m^2^)	28 (24, 32)	29 (24, 33)	28 (25, 32)	27 (24, 32)	0.4
Systolic BP (mmHg)	126 (115, 139)	125 (113, 137)	127 (116, 140)	130 (117, 141)	0.5
Diastolic BP (mmHg)	68 (60, 75)	69 (61, 77)	68 (60, 73)	65 (57, 73)	0.15
Total Cholesterol (mmol/L)	4.84 (4.03, 5.64)	4.86 (4.09, 5.72)	4.84 (4.09, 5.46)	4.60 (3.78, 5.59)	0.5
Direct HDL cholesterol (mmol/L)	1.29 (1.03, 1.68)	1.32 (1.01, 1.68)	1.29 (1.03, 1.63)	1.37 (1.09, 1.81)	0.3
LDL cholesterol (mmol/L)	2.79 (2.30, 3.28)	2.92 (2.40, 3.26)	2.78 (2.23, 3.31)	2.49 (2.18, 3.21)	0.084
Triglyceride (mmol/L)	1.38 (1.05, 1.81)	1.36 (0.99, 1.83)	1.43 (1.13, 1.84)	1.32 (1.05, 1.69)	0.3
Glycohaemoglobin (%)	5.70 (5.40, 6.10)	5.60 (5.30, 5.90)	5.80 (5.50, 6.10)	5.80 (5.50, 6.20)	0.011
Creatinine (umol/L)	82 (68, 97)	82 (69, 95)	84 (68, 103)	81 (68, 101)	0.7
eGFR (mL/min/1.73 m²)	73 (57, 84)	76 (61, 85)	67 (53, 80)	73 (57, 85)	0.2
Albumin (g/dL)	4.20 (4.00, 4.40)	4.30 (4.10, 4.40)	4.30 (4.00, 4.40)	4.10 (4.00, 4.30)	0.2
Total calcium (mmol/L)	2.35 (2.30, 2.43)	2.35 (2.30, 2.43)	2.35 (2.30, 2.43)	2.35 (2.30, 2.40)	0.5
Adjusted calcium (mmol/L)	2.19 (2.04, 2.33)	2.19 (2.04, 2.32)	2.17 (2.01, 2.34)	2.22 (2.09, 2.38)	0.4
General health					0.2
Excellent	2 604 303 (11%)	1 330 787 (11%)	576 431 (9.4%)	697 085 (15%)	
Very good	6 249 475 (27%)	3 794 553 (30%)	1 533 138 (25%)	921 783 (20%)	
Good	8 541 237 (37%)	4 890 913 (39%)	2 335 147 (38%)	1 315 178 (29%)	
Fair	4 167 290 (18%)	1 790 973 (14%)	1 114 528 (18%)	1 261 789 (28%)	
Poor	1 564 121 (6.8%)	696 313 (5.6%)	553 888 (9.1%)	313 919 (7.0%)	
Alcohol consumption					0.6
None	8 383 323 (36%)	4 481 499 (36%)	2 316 306 (38%)	1 585 519 (35%)	
Moderate	9 936 127 (43%)	5 788 114 (46%)	2 214 617 (36%)	1 933 396 (43%)	
Heavy	4 806 975 (21%)	2 233 926 (18%)	1 582 208 (26%)	990 840 (22%)	
Smoking					0.13
Never	10 123 816 (44%)	5 859 376 (47%)	2 619 938 (43%)	1 644 501 (36%)	
Former	3 916 354 (17%)	2 322 811 (19%)	1 121 266 (18%)	472 277 (10%)	
Current	9 086 256 (39%)	4 321 353 (35%)	2 371 927 (39%)	2 392 976 (53%)	
Hypertension	13 816 026 (60%)	6 702 666 (54%)	4 274 600 (70%)	2 838 760 (63%)	0.064
Hyperlipidaemia	13 046 169 (56%)	6 344 064 (51%)	3 441 829 (56%)	3 260 277 (72%)	0.038
Diabetes mellitus	4 007 241 (17%)	1 993 034 (16%)	886 309 (14%)	1 127 899 (25%)	0.4
Baseline CVD	4 457 196 (19%)	1 664 990 (13%)	1 479 506 (24%)	1 312 700 (29%)	0.051
Congestive heart failure	1 627 752 (7.0%)	698 001 (5.6%)	459 684 (7.5%)	470 067 (10%)	0.3
Coronary heart disease	1 567 903 (6.8%)	617 763 (4.9%)	270 870 (4.4%)	679 270 (15%)	0.023
Angina/angina pectoris	568 628 (2.5%)	330 828 (2.6%)	155 988 (2.6%)	81 812 (1.8%)	0.8
Heart attack	1 409 918 (6.1%)	416 708 (3.3%)	490 451 (8.0%)	502 759 (11%)	0.035
Stroke	1 505 386 (6.5%)	476 351 (3.8%)	513 965 (8.4%)	515 070 (11%)	0.2
Time from cancer diagnosis (years)	26 (20, 31)	24 (19, 28)	26 (21, 32)	31 (27, 33)	<0.001
Number of cancers					0.14
1	20 521 456 (89%)	11 604 480 (93%)	5 142 359 (84%)	3 774 616 (84%)	
2	2 377 598 (10%)	899 059 (7.2%)	775 843 (13%)	702 695 (16%)	
3	105 615 (0.5%)	0 (0%)	73 172 (1.2%)	32 443 (0.7%)	
4	121 758 (0.5%)	0 (0%)	121 758 (2.0%)	0 (0%)	

^a^Median (Q1, Q3); *n* (%).

^b^Design-based Kruskal–Wallis test; Pearson’s *χ*²: Rao andScott adjustment.

Participants were categorized into three groups based on their AAC-24 scores: no AAC (AAC24 = 0, *n*= 12 503 539), moderate AAC (AAC24 = 1–6, *n* = 6 113 131), and severe AAC (AAC24 > 6, *n* = 4 509 754) (*Table 1*). The distribution of the AAC-24 score values in both moderate and severe AAC groups is detailed in (see Supplementary material online, *Table S4*). Cancer survivors with no AAC (AAC24 scores = 0) comprised the majority (54%) of the cohort (*Table 1*). Those with severe AAC (19.5%) were significantly older, with a median age of 74 years, compared to 69 years in the moderate AAC group and 62 years in the no-AAC group. The severe AAC group also had a higher prevalence of CV risk factors and comorbid conditions, including hypertension (63%), hyperlipidaemia (72%), and baseline CVD (29%). BMI was comparable across groups, with an overall median of 28 kg/m². Systolic blood pressure was slightly higher in the severe AAC group (130 mmHg) compared to the moderate (127 mmHg) and none (125 mmHg) groups. The distribution of demographics, comorbidities, and CV risk factors between those groups showed significant differences across several characteristics, as outlined in (*Table 1*).

**Figure 1:**
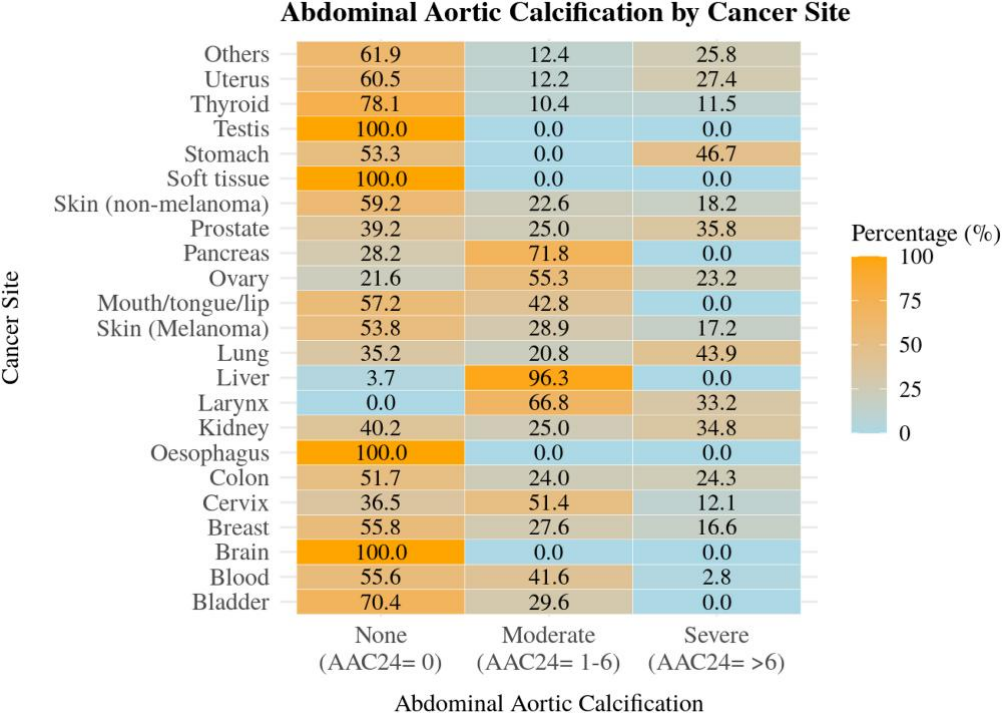
Survey-weighted heatmap of abdominal aortic calcification distribution by cancer site in NHANES (2013–2018) cancer copulation.

*Figure 1* illustrates the distribution of AAC across various cancer sites. Severe AAC (AAC24 scores >6) was most prevalent among survivors of stomach cancer (46.7%), followed by lung cancer (43.9%). Among urological malignancies, prostate (35.8%) and kidney (34.8%) cancer survivors also exhibited a substantial burden of severe AAC. Moderate AAC (AAC24 scores = 1–6) was most frequently observed in liver cancer survivors (96.3%), followed by those with pancreatic (71.8%) and ovarian (55.3%) cancers. In contrast, survivors of testicular, soft tissue, oesophageal, and brain cancers had no evidence of vascular calcification, with all individuals in these groups classified as AAC-free (AAC24 = 0).

### Outcomes

Over a median follow-up of 69 months, 4 199 131 all-cause deaths (18%) and 1 160 618 CV deaths (5.0%) were recorded in the weighted dataset. Mortality rates were highest among individuals with severe AAC (33% all-cause, 12% CV mortality), compared to moderate AAC (26% all-cause, 7.9% CV mortality) and no AAC (17% all-cause, 4.2% CV mortality).

Over up to 7 years of follow-up, we observed a decreased survival among cancer survivors with higher AAC-24 scores compared to those with lower scores or no AAC. At 1 year, survival was similar between those with severe AAC and no AAC (99.1% vs. 99.9%). However, by 5 years, survival was lower in the severe AAC group (88.8% vs. 94.4%), and this gap widened further at 7 years (71.0% vs. 88.4%). The survival figures stratified by AAC severity categories are detailed in (*Table 2*).

**Table 2:** Unadjusted cumulative survival of cancer patients stratified by AAC-24 scores used to define abdominal aortic calcification at 1, 3, 5, and 7 years^a^

Time (years)	No AAC (AAC24 = 0)^b^ *n* = 12 503 539	Moderate AAC (AAC24 = 1–6)^b^ *n* = 6 113 131	Severe AAC (AAC24 > 6)^b^ *n* = 4 509 754
1 year	99.9% (99.7–100.0)	99.6% (98.9–100.0)	99.1% (97.7–100.0)
3 years	97.4% (95.4–99.5)	98.0% (96.5–99.6)	96.0% (92.8–99.4)
5 years	94.4% (91.3–97.6)	95.3% (92.2–98.4)	88.8% (83.4–94.6)
7 years	88.4% (83.1–94.0)	84.7% (80.3–89.5)	71.0% (61.3–82.2)

^a^All analyses and estimates are based on weighted records.

^b^Survival % (95% CI).

**Figure 2:**
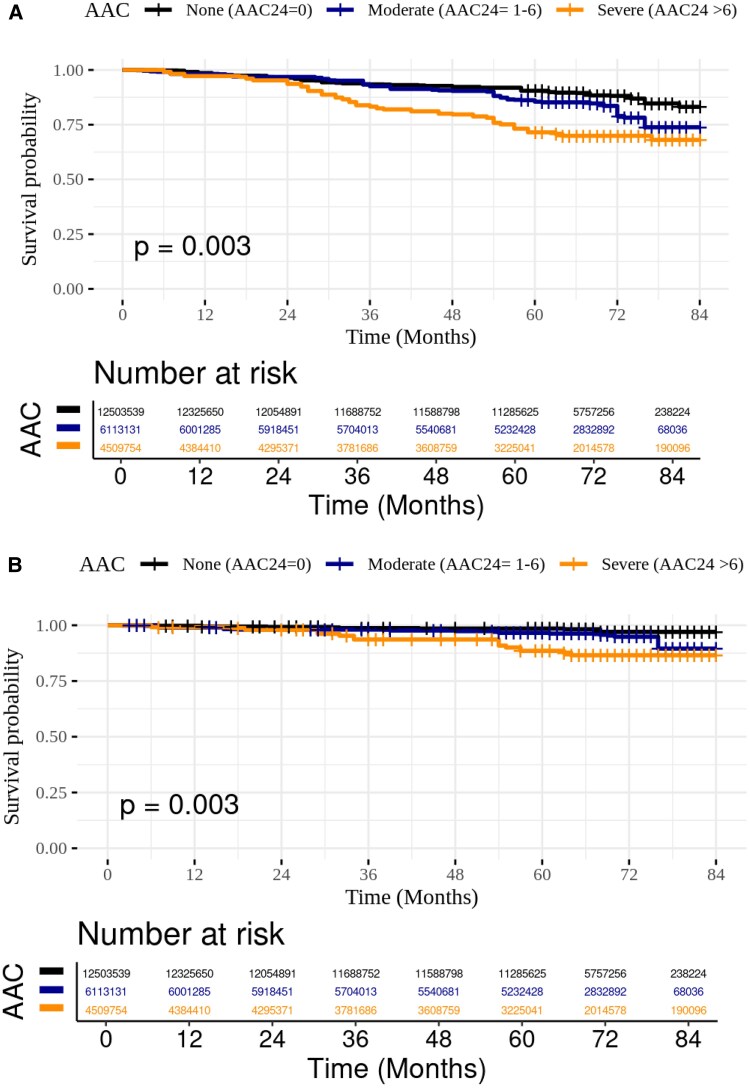
Survey-weighted Kaplan–Meier (KM) survival curves by abdominal aortic calcification severity in NHANES (2013–2018) cancer population for (*A*) all-cause mortality and (*B*) CV mortality. *P*-value for log-rank (<0.01).

*Figure 2* presents the weighted Kaplan–Meier survival curves for all-cause mortality among cancer survivors, stratified by AAC-24 severity categories. Survivors with severe AAC had lower survival probabilities than those with moderate or no AAC. Survival disparities widened over time, with the greatest differences emerging at longer follow-up durations. We observed an association between AAC severity and survival outcomes; compared to survivors with no AAC (AAC-24 = 0), those with moderate AAC (AAC24 = 1–6) had a hazard ratio (HR) of 1.00 for all-cause mortality [95% confidence interval (CI): 0.59–1.69, *P* = 0.98] and an subdistribution hazard ratio (sHR) of 1.27 for CV mortality (95% CI: 1.03–1.57, *P* = 0.02). Individuals with severe AAC (AAC24 > 6) had an HR of 1.80 for all-cause mortality (95% CI: 1.01–3.21, *P* = 0.04) and an sHR of 1.39 for CV mortality (95% CI: 1.09–1.76, *P* = 0.006).

In the continuous models, higher AAC-24 scores were associated with increased risks of all-cause and CV mortality (*Table 3*). In the fully adjusted model, each unit increase in AAC-24 score was associated with a 4% higher risk of all-cause mortality in Cox proportional hazard (HR: 1.04; 95% CI: 1.00–1.09; *P* = 0.047) and an 7% increased risk of CV mortality in the Fine and Gray models (sHR: 1.07; 95% CI: 1.02–1.12; *P* = 0.002).

**Table 3:** Survey-weighted association between abdominal aortic calcification score and outcomes

AAC-24 score (continuous variable)	All-cause mortality			Cardiovascular mortality		
Per one-unit increase in score	HR^a^	95% CI^a^	*P*-value	sHR^a^	95% CI^a^	*P*-value
Model 1 (unadjusted)	1.07	1.03, 1.12	<0.001	1.13	1.08, 1.18	<0.001
Model 2^b^ (partially adjusted)	1.06	1.02, 1.11	0.002	1.08	1.03, 1.13	<0.001
Model 3^c^ (fully adjusted)	1.04	1.00, 1.09	0.047	1.07	1.02, 1.12	0.002
Model 4^d^ (by severity group)						
None (AAC24 = 0)	Reference	−	−	Reference	−	−
Moderate (AAC24 = 1–6)	1.00	0.59, 1.69	0.98	1.27	1.03, 1.57	0.02
Severe (AAC24 > 6)	1.80	1.01, 3.21	0.04	1.39	1.09, 1.76	0.006

HR, hazard Ratio; sHR, subdistribution hazard ratio; CI, confidence interval.

^a^Model 1: unadjusted.

^b^Model 2: adjusted for sex, age, education, income, hypertension, diabetes mellitus, hyperlipidaemia, smoking, and BMI.

^c^Model 3: included adjustments for Model 2 variables plus alcohol intake, marital status, HDL, TG, cancer site, time from cancer diagnosis, baseline CVD, general health perception.

^d^Adjusted for all variables included in Model 3.

### Calibration and discrimination of models

Overall, the fully adjusted models were reasonably well calibrated on the original sample but showed some degree of overfitting in the bootstrap samples during internal validation, as indicated by the calibration slope (optimism-adjusted C-slope = 0.80 for all-cause mortality and 0.87 for CV mortality). The discriminative performance of the fully adjusted models was also acceptable to good. The C-statistic for the fully adjusted models was 0.74 (optimism-adjusted: 0.70) for all-cause mortality and 0.68 (optimism-adjusted: 0.67) for CV mortality (*Table 4*).

**Table 4:** Predictive performance of the fitted models

All-cause mortality	Discrimination (C-statistic)		Calibration (C-slope)	
Per one-unit increase in score (Models 1–3)	Original	Optimism-adjusted	Original	Optimism- adjusted
Model 1 (unadjusted)	0.58	0.58	1	1.19
Model 2^a^ (partially adjusted)	0.71	0.68	1	0.86
Model 3^b^ (fully adjusted)	0.74	0.70	1	0.80
Model 4^c^ (by severity group)	0.74	0.70	1	0.79
CV mortality				
Model 1 (unadjusted)	0.54	0.54	1	1.09
Model 2^a^ (partially adjusted)	0.65	0.64	1	0.92
Model 3^b^ (fully adjusted)	0.68	0.67	1	0.87
Model 4^c^ (by severity group)	0.68	0.66	1	0.87

Model 1: unadjusted.

^a^Model 2: adjusted for sex, age, education, income, hypertension, diabetes mellitus, hyperlipidaemia, smoking, BMI.

^b^Model 3: included adjustments for Model 2 variables plus alcohol intake, marital status, HDL, TG, cancer site, time from cancer diagnosis, baseline CVD, general health perception.

^c^Adjusted for all variables included in Model 3.

### Supplementary analysis

Supplementary material online, *Table S5* presents the results after excluding cancers without representation across all three AAC strata (testis, stomach, pancreas, mouth/tongue/lip, liver, larynx, oesophagus, brain, and bladder) from the fully adjusted models. In this restricted cohort of 448 unweighted participants (corresponding to 21 660 931 weighted records), the associations between AAC-24 and mortality outcomes remained broadly consistent with the main analysis. Each unit increase in AAC-24 score was associated with a 5% higher risk of all-cause mortality (HR: 1.05, 95% CI: 1.00–1.10, *P* = 0.04) and an 8% higher risk of CV mortality (sHR: 1.08, 95% CI: 1.01–1.14, *P* = 0.02). By severity group, only survivors with severe AAC (AAC24 > 6) had a significantly higher risk of all-cause mortality (HR: 1.89, 95% CI: 1.04–3.44, *P* = 0.04) and CV mortality (sHR: 2.81, 95% CI: 1.17–6.76, *P* = 0.02) compared with those with no AAC.

### Sensitivity analysis

A sensitivity analysis excluding participants with baseline CVD and hypercalcaemia (>2.6 mmol/L) was conducted to test the robustness of findings in this subpopulation. Survey-weighted differences in baseline characteristics and cancer sites by AAC categories are presented in (see Supplementary material online, *Table S6* and *Figure 2*, respectively).

In this subset of 17 492 167 weighted records, the association between AAC-24 scores and mortality outcomes remained consistent, with higher AAC scores continuing to predict increased risks of all-cause and CV mortality. Kaplan–Meier survival analyses also showed a similar pattern of survival decline across AAC severity categories (see Supplementary material online, *Figure S3*). Compared to those with no AAC (AAC-24 = 0), survivors with moderate AAC (AAC-24 = 1–6) had numerically insignificant increased risk of all-cause mortality (HR: 1.68, 95% CI: 0.78–3.60, *P* = 0.17) and CV mortality (sHR: 1.13, 95% CI: 0.86–1.49, *P* = 0.37). However, survivors with severe AAC (AAC24 > 6) had a significantly higher risk of all-cause mortality (HR: 2.73, 95% CI: 1.22–6.10, *P* = 0.01) and CV mortality (sHR: 1.49, 95% CI: 1.03–2.15, *P* = 0.03).

For all-cause mortality, each unit increase in AAC-24 was associated with a 7% higher risk of all-cause mortality (HR: 1.07, 95% CI: 1.01–1.14, *P* = 0.009). This association remained significant after adjustments for sex, age, education, income, hypertension, diabetes mellitus, hyperlipidaemia, smoking, and BMI in Model 2, yielding an adjusted hazard ratio (aHR) of 1.06 (95% CI: 1.01–1.12, *P* = 0.01). In Model 3, which further adjusted for alcohol intake, marital status, HDL, triglycerides, cancer site, time from cancer diagnosis, baseline CVD, and general health perception, the association persisted with an aHR of 1.05 (95% CI: 1.00–1.11, *P* = 0.042). Similarly, for CV mortality, each unit increase in AAC-24 was associated with an unadjusted sHR of 1.12 (95% CI: 1.02–1.24, *P* = 0.01). This association remained significant in Model 2 (sHR 1.10, 95% CI: 1.02–1.19, *P* = 0.007) and Model 3 (sHR 1.07, 95% CI: 1.00–1.14, *P* = 0.045), indicating a consistent, increased risk even after extensive adjustments for potential confounders (see Supplementary material online, *Table S7*). The discrimination and calibration of the fitted models in this sensitivity analysis showed similar trends to the primary analysis, with reasonable calibration and acceptable discriminative performance across models after adjustment for in-sample optimism (see Supplementary material online, *Table S8*).

## Discussion

There were three important findings in this study. First, the prevalence of AAC was substantial among cancer survivors, with over 45% of the cohort exhibiting AAC. Among them, nearly one in five individuals (19.5%) had severe AAC (AAC-24 score >6), highlighting a considerable burden of advanced vascular calcification in this population. Second, the distribution of AAC varied by cancer type, with severe AAC most prevalent among survivors of stomach, lung, prostate, and kidney cancers. Finally, when analysed as a continuous predictor, higher AAC-24 scores were independently associated with an increased risk of all-cause and CV mortality in the cancer population, beyond demographic, socioeconomic, traditional CV risk factors, baseline comorbid CVDs, and cancer-related characteristics. The significance of these findings persisted even when the analysis was restricted to cancer types with representation across all AAC strata and when participants with comorbid CVDs or evidence of hypercalcaemia were excluded, suggesting that AAC scores have the potential to serve as an independent prognostic marker in cancer survivorship.

DXA has emerged as a valuable imaging tool for detecting AAC, a marker of systemic atherosclerosis and subclinical CVD.^35^ Originally designed for bone mineral density assessment, DXA now provides a widely accessible, low-radiation alternative for quantifying AAC, making it a particularly useful method for CV risk stratification in cancer survivors. Compared to computed tomography (CT), which offers high-resolution visualization of arterial calcification, DXA presents a cost-effective and practical option for large-scale clinical and epidemiological studies. Our study used Kauppila’s AAC-24 scoring system on DXA scans to quantify AAC burden, building upon prior studies in other populations that found AAC detected via DXA is predictive of all-cause mortality and CV events.^7,36–38^ As such, our findings support the role of AAC detected on DXA in long-term CV risk stratification in cancer survivorship care, particularly in settings where DXA is already in routine use for bone health monitoring. This raises the possibility of opportunistic AAC screening in cardio-oncology services.

In our study, cancer survivors with severe AAC were significantly older (median age: 74 years) than those with moderate (69 years) or no AAC (62 years), consistent with previous reports indicating that AAC prevalence and extent increase with age.^39^ Both moderate and severe AAC groups exhibited a higher burden of traditional CV risk factors and comorbid CVD compared to those without AAC. In the severe AAC group, our study identified a high prevalence of current smoking (53%), diabetes mellitus (25%), hypertension (63%), and hyperlipidaemia (72%) among cancer survivors with severe AAC, further supporting the well-established link between AAC and traditional CV risk factors.^40^ Ethnic disparities in AAC prevalence and risk factors have been previously highlighted, particularly in the Multi-Ethnic Study of Atherosclerosis (MESA), which found that European Americans exhibited the highest AAC prevalence compared to Hispanics, African Americans, and Chinese Americans.^41^ In our study, the distribution of moderate and severe AAC in cancer reflected similar racial trends, with most cancer survivors in these categories being non-Hispanic White. Moreover, Allison *et al*.^41^ have shown that risk factors for AAC in the general population differ by ethnicity—for instance, hypertension and cholesterol medication use are key contributors in European Americans, whereas cholesterol plays a more prominent role in Hispanics. Such findings emphasize the need for further research into ethnic-specific differences in risk profiles for AAC burden among cancer survivors.

AAC is a complex pathological process driven by osteogenic transformation of vascular smooth muscle cells (VSMCs). The progression of AAC is influenced by chronic inflammation, oxidative stress, and dysregulated mineral metabolism, all of which contribute to the loss of vascular elasticity, endothelial dysfunction, and increased CV risk.^40^ Inflammatory signalling plays a key role in this process by stimulating VSMCs to undergo osteogenic differentiation, promoting vascular calcification, and accelerating the transition from adaptive remodelling to pathological atherosclerosis.^42,43^

In the current study, we observed a 4 and 7% increase in the risk of all-cause and CV mortality, respectively, with each unit increase in AAC in cancer survivors. Prior research has demonstrated that AAC is independently associated with mortality and CV events. In a meta-analysis of 10 longitudinal studies in the general population, AAC was regarded as an independent strong predictor of CV risk events or death.^44^ In 2021, a larger systematic review and meta-analysis of 52 population-based studies restricted to patients with chronic kidney disease and the elderly general population similarly reported that individuals with any AAC had a significantly higher risk of mortality compared to those without AAC, with a pooled HR of 1.72 (95% CI: 1.51–1.95) for all-cause mortality and 1.86 (95% CI: 1.57–2.19) for CV mortality.^2^ Our study aligns with these findings, establishing the prognostic value of AAC for long-term risk stratification in cancer survivors.

In contrast, more recent studies examining the association between AAC and outcomes in the NHANES general population have reported non-significant associations with CV mortality after adjusting for traditional CV risk factors,^15,16^ raising questions about the independent prognostic role of AAC in the US general population. However, in our study, the associations between AAC and CV mortality in the US cancer survivors remained significant even after sequential adjustments for demographic variables, socioeconomic factors, traditional CV risk factors, and additional confounders, including pre-existing CVD and cancer-specific characteristics. The persistence of this association suggests that AAC, as a marker of systemic atherosclerosis, may carry a stronger prognostic impact in high-risk populations, like cancer survivors, in whom evidence suggests that atherosclerosis develops earlier and is more prevalent than in the general population.^17^ While this excess risk in cancer survivors is partly due to shared risk factors between cancer and CVD, including hypertension, obesity, and chronic inflammation, which are implicated in both carcinogenesis and atherosclerosis. Several cancer therapy-related pathways may also contribute to cardiovascular dysfunction and the development of vascular calcification in this population. Anthracyclines generate reactive oxygen species, leading to endothelial dysfunction and vascular permeability. Plant alkaloids further damage the endothelium by increasing vascular permeability, while alkylating agents induce oxidative stress and thrombosis, promoting arteriosclerosis and vascular fibrosis.^45^ Tyrosine kinase inhibitors with anti-VEGF properties have been associated with a higher incidence of thrombotic and CV events. Immune checkpoint inhibitors have been associated with increased atherosclerotic progression and increased CV events.^46,47^ They promote a chronic pro-inflammatory state by stimulating the release of cytokines, which enhance monocyte adhesion, foam cell formation, and endothelial injury.^48^ Radiation therapy has similarly been linked to ischaemic cardiovascular events in cancer survivors, with proposed mechanisms including endothelial damage, arterial fibrosis, coronary arteriosclerosis, and a prothrombotic state.^49^ This may further explain the higher prevalence of systemic atherosclerosis in cancer survivors and the stronger prognostic significance of its markers in this population compared to the general population.

Arterial calcification has traditionally been considered an irreversible endpoint of atherosclerotic disease.^39^ Growing evidence indicates that it can be slowed or even reversed, with several therapies being explored in clinical trials to inhibit its progression. In patients on haemodialysis, bisphosphonates such as etidronate have been shown to inhibit and even reverse the progression of AAC. Cinacalcet, a calcimimetic used in secondary hyperparathyroidism, has demonstrated the most promising results among current pharmacological options, with a network meta-analysis identifying it as the most effective therapy for vascular calcification in this patient group.^50^ Similarly, nifedipine was shown to reduce the number of newly formed coronary lesions in early-stage coronary artery disease, and to slow down coronary artery calcification when compared to coamizolide in patients with hypertension.^51^ Beyond pharmacologic approaches, lifestyle and metabolic factors also play a key role in modifying AAC progression. Higher cardiovascular health, measured by composite metrics like Life’s Essential 8, is inversely associated with AAC severity, highlighting the importance of smoking cessation, blood pressure control, glycaemic management, and lipid regulation. Additionally, diets rich in fruits, vegetables, and low sodium, combined with regular physical activity, are linked to a lower vascular calcification burden.^52^ AAC management should not be approached in isolation but rather as part of a comprehensive cardiovascular prevention strategy, particularly in high-risk groups such as cancer survivors.

### Strengths and limitations

Our study differs from prior research in several ways. First, it is the first to assess the prognostic utility of AAC scores in cancer survivors using a large, nationally representative cohort. The use of NHANES data ensured a well-powered, weighted sample, enhancing generalizability to US cancer survivors aged ≥40 years. Second, mortality data were obtained from a reliable nationwide registry, ensuring robust follow-up. Third, DXA—a cost-effective, widely accessible imaging modality—was used to quantify AAC, making our approach particularly relevant for large-scale clinical screening and epidemiological studies in cancer survivors. Future research should compare DXA-based AAC assessment with other imaging modalities in cardio-oncology settings.

Nonetheless, this study has several limitations. First, its observational design precludes causal inference, and while extensive adjustments were made, residual confounding cannot be ruled out. Second, AAC was assessed only at baseline, preventing evaluation of its progression over time. Third, we lacked data on cancer stage, grade, and oncological treatments, limiting insight into how specific therapies influence AAC progression and its association with mortality. Fourth, DXA-based AAC assessment cannot differentiate between intimal and medial calcifications, which may have distinct pathophysiological and prognostic implications. Additionally, we did not assess whether calcifications in other aortic segments detected on DXA contribute to mortality risk. Fifth, the absence of comparative AAC measurements using other imaging modalities, such as CT or MRI, restricts direct comparisons with established techniques. Sixth, the potential confounding effect of LDL cholesterol was not examined; however, prior UK Biobank findings suggest AAC is not significantly correlated with LDL cholesterol, mitigating concerns about its impact.^53^ Seventh, Similar to other survey-based research studies, information regarding cancer diagnosis, comorbidities, risk factors, and prior treatment is reliant on participants correctly entering this information, without a way of cross-referencing the information with medical records; this may introduce recall bias and misclassifications. Eighth, the NHANES does not provide AAC scores for participants aged <40 years, limiting generalizability to younger cancer populations. Ninth, while sampling weighting is essential to account for the survey’s multistage sampling design and non-response, the use of these weights in outcome modelling may introduce sampling variability and should be interpreted with appropriate caution. Finally, while we observed variation in AAC burden across cancer types, we were unable to conduct stratified subgroup analyses to formally assess which cancer subgroups might benefit most from AAC screening, as some had an insufficient number of events per variable (EPV) to ensure adequately powered results.

Despite these limitations, our study provides a valuable contribution to the literature. Given its prognostic utility, DXA holds promise as a potential screening tool for vascular health assessment in cardio-oncology care. Future studies should validate these findings across diverse populations and explore AAC score integration into comprehensive cardio-oncology risk models to improve long-term CV risk stratification in cancer survivorship.

## Conclusions

The extent of AAC detected on lateral lumbar spine DXA scans among US cancer survivors was associated with an increased risk of all-cause and CV mortality. The present findings highlight the importance of routine evaluation of AAC on lateral lumbar spine DXA scans to identify subclinical atherosclerosis and stratify CV risk in cancer survivors.

## Lead author biography


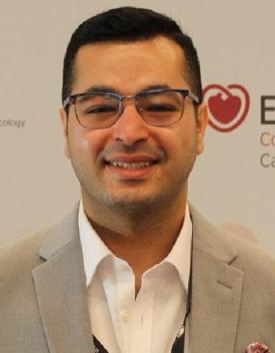


Dr Mustafa Al-jarshawi is an Academic Clinical Fellow in Cardiology at Keele University, currently undertaking integrated academic specialty training in Cardiology at the Royal Stoke University Hospital in the West Midlands, UK. Funded by the UK’s National Institute for Health and Care Research, his work focuses on big data applications in cardiology, drawing on large-scale electronic health records from the UK and USA. His current primary research interest lies in cardiovascular risk stratification and the prognostic utility of novel and non-invasive biomarkers in special populations, with the aim of informing long-term outcomes and real-world clinical care.

## Supplementary Material

oeaf116_Supplementary_Data

## Data Availability

The data underlying this article are available in the NHANES website, at https://wwwn.cdc.gov/nchs/nhanes/Default.aspx.
