# Supplementary material for: Prognostic significance of abdominal aortic calcification scores on dual-energy X-ray absorptiometry scans for mortality in cancer survivors: NHANES-based cohort study (2013–2019)
Source: Eur Heart J Open. 2025 Sep 1;5(5):oeaf116. doi: 10.1093/ehjopen/oeaf116 (PMC12492483; doi:10.1093/ehjopen/oeaf116)
Supplement: oeaf116_Supplementary_Data [file oeaf116_supplementary_data.docx]

**Supplementary figure 1:** Study flowchart


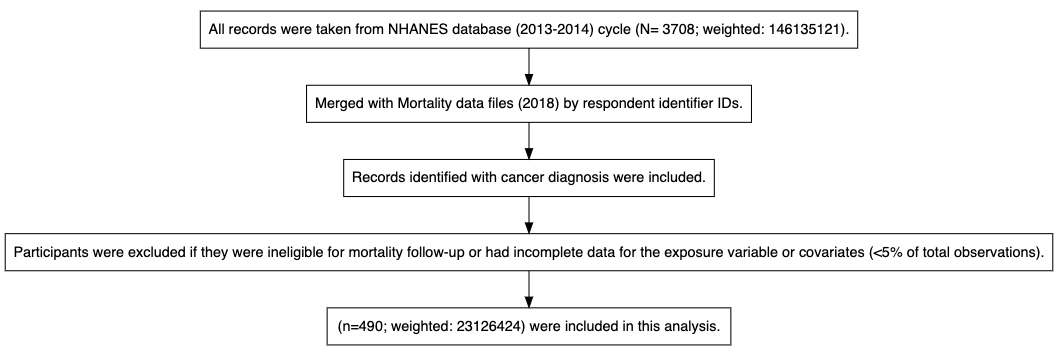


**Supplementary table 1: Weighted sample size adequacy calculations for prognostic modelling in this study**

| **Outcome** | **Minimum weighted sample size required** | **Shrinkage** | **Parameter** | **CS_Rsq*** | **Max_Rsq** | **Nag_Rsq** | **EPP** |
| --- | --- | --- | --- | --- | --- | --- | --- |
| **Primary** (all-cause mortality) | 2189 | 0.900 | 55 | 0.2 | 0.864 | 0.231 | 37.97 |
| **Secondary** (CV mortality) | 2189 | 0.900 | 55 | 0.2 | 0.709 | 0.282 | 10.55 |

NB: Assuming 0.05 margin of error in estimation of overall risk over 7-year follow-up period. Events per Predictor Parameter (EPP) assumes overall event rate = 0.18 (for all-cause mortality) & 0.05 (for CV mortality) as observed in our NHANES cancer population. * R-squared was set at a conservative value to reflect anticipated model performance. NB: Assuming 0.05 acceptable difference in apparent & adjusted R-squared.

**Supplementary table 2: Schoenfeld residual test results for proportional hazards assumption**

| **Variable** | **Chi-Square (χ²)** | **p-value** |
| --- | --- | --- |
| **Sex** | 0.2896 | 0.590 |
| **HDL** | 0.1834 | 0.668 |
| **TG** | 0.1799 | 0.671 |
| **Cancer_type** | 0.9230 | 0.337 |
| **Marital_status** | 0.5473 | 0.459 |
| **SBP** | 0.1811 | 0.670 |
| **Cancer_Duration** | 0.0457 | 0.831 |
| **Gen_health** | 0.0822 | 0.774 |
| **Education_cat** | 0.5655 | 0.452 |
| **Income_cat** | 5.0045 | 0.025 |
| **Alcohol_Status** | 0.7178 | 0.397 |
| **Age** | 0.6931 | 0.405 |
| **AAC24** | 2.4976 | 0.114 |
| **BMI** | 4.2095 | 0.040 |
| **Hypertension** | 7.5628 | 0.006 |
| **DM** | 0.1166 | 0.733 |
| **Smoking_Status** | 0.2175 | 0.641 |
| **Hyperlipidaemia** | 2.4315 | 0.119 |
| **CVD** | 0.4575 | 0.499 |
| **GLOBAL** | 29.5397 | 0.058 |

**Supplementary table 3: Variance inflation factor for covariates included in analysis**

| **Covariate** | **VIF^+^** |
| --- | --- |
| **Sex** | 1.3661 |
| **Education_cat** | 1.1764 |
| **Income_cat** | 1.2487 |
| **Marital_status** | 1.0884 |
| **BMI** | 1.2252 |
| **SBP** | 1.1444 |
| **Hypertension** | 1.1996 |
| **DM** | 1.0894 |
| **Smoking_Status** | 1.1282 |
| **Hyperlipidaemia** | 1.0645 |
| **HDL** | 1.5128 |
| **TG** | 1.4326 |
| **Gen_health** | 1.2880 |
| **CVD** | 1.1293 |
| **Cancer_type** | 1.1310 |
| **Cancer_Duration** | 1.1383 |
| **Age*** | - |

^+^Variance inflation factor. *Reference variable in analysis.

| Supplementary table 4: Distribution of AAC-24 scores in moderate and severe abdominal aortic calcification by corresponding lumbar vertebral level L1–L4 of Aorta on DXA scan lateral lumbar images | | | | |
| --- | --- | --- | --- | --- |
| **Region**  **Sub-region scores** | **Moderate**  **(AAC24= 1-6)**  N = 6,113,131^1^ | **Severe**  **(AAC24 >6)**  N = 4,509,754^1^ | | **p-value**^2^ |
| **AAC L1 Posterior score** |  | |  | <0.001 |
| 0 | 5,152,198 (84%) | | 1,925,422 (43%) |  |
| 1 | 737,361 (12%) | | 1,610,971 (36%) |  |
| 2 | 223,573 (3.7%) | | 504,501 (11%) |  |
| 3 | 0 (0%) | | 468,860 (10%) |  |
| **AAC L1 Anterior score** |  | |  | <0.001 |
| 0 | 5,838,134 (96%) | | 3,299,968 (73%) |  |
| 1 | 191,027 (3.1%) | | 773,979 (17%) |  |
| 2 | 83,970 (1.4%) | | 326,416 (7.2%) |  |
| 3 | 0 (0%) | | 109,392 (2.4%) |  |
| **AAC L2 Posterior score** |  | |  | <0.001 |
| 0 | 4,072,467 (67%) | | 1,433,878 (32%) |  |
| 1 | 1,824,809 (30%) | | 1,759,823 (39%) |  |
| 2 | 116,322 (1.9%) | | 410,545 (9.1%) |  |
| 3 | 99,534 (1.6%) | | 905,508 (20%) |  |
| **AAC L2 Anterior score** |  | |  | <0.001 |
| 0 | 5,093,072 (83%) | | 1,928,296 (43%) |  |
| 1 | 882,938 (14%) | | 1,769,054 (39%) |  |
| 2 | 137,121 (2.2%) | | 680,513 (15%) |  |
| 3 | 0 (0%) | | 131,892 (2.9%) |  |
| **AAC L3 Posterior score** |  | |  | <0.001 |
| 0 | 3,412,303 (56%) | | 320,484 (7.1%) |  |
| 1 | 2,403,314 (39%) | | 1,755,541 (39%) |  |
| 2 | 181,197 (3.0%) | | 1,050,041 (23%) |  |
| 3 | 116,317 (1.9%) | | 1,383,688 (31%) |  |
| **AAC L3 Anterior score** |  | |  | <0.001 |
| 0 | 4,185,722 (68%) | | 516,266 (11%) |  |
| 1 | 1,783,278 (29%) | | 1,349,589 (30%) |  |
| 2 | 126,763 (2.1%) | | 824,920 (18%) |  |
| 3 | 17,368 (0.3%) | | 1,818,979 (40%) |  |
| **AAC L4 Posterior score** |  | |  | <0.001 |
| 0 | 2,802,765 (46%) | | 505,610 (11%) |  |
| 1 | 2,713,676 (44%) | | 1,569,609 (35%) |  |
| 2 | 368,990 (6.0%) | | 678,653 (15%) |  |
| 3 | 227,700 (3.7%) | | 1,755,882 (39%) |  |
| **AAC L4 Anterior score** |  | |  | <0.001 |
| 0 | 4,154,093 (68%) | | 1,075,425 (24%) |  |
| 1 | 1,726,370 (28%) | | 2,371,582 (53%) |  |
| 2 | 182,252 (3.0%) | | 436,209 (9.7%) |  |
| 3 | 50,416 (0.8%) | | 626,539 (14%) |  |
| **AAC L4 mid score** |  | |  | <0.001 |
| 0 | 4,835,799 (79%) | | 1,966,805 (44%) |  |
| 1 | 1,077,078 (18%) | | 1,636,163 (36%) |  |
| 2 | 200,254 (3.3%) | | 906,787 (20%) |  |
| **AAC Anterior 8 score** |  | |  | <0.001 |
| 0 | 3,163,251 (52%) | | 959,554 (21%) |  |
| 1 | 2,384,850 (39%) | | 1,571,658 (35%) |  |
| 2 | 565,030 (9.2%) | | 1,745,985 (39%) |  |
| 3 | 0 (0%) | | 204,831 (4.5%) |  |
| 4 | 0 (0%) | | 27,727 (0.6%) |  |
| **AAC Posterior 8 score** |  | |  | <0.001 |
| 0 | 1,023,334 (17%) | | 596,850 (13%) |  |
| 1 | 4,554,989 (75%) | | 1,300,725 (29%) |  |
| 2 | 431,454 (7.1%) | | 1,503,900 (33%) |  |
| 3 | 68,991 (1.1%) | | 865,938 (19%) |  |
| 4 | 34,363 (0.6%) | | 242,341 (5.4%) |  |
| ^1^Median (Q1, Q3); n (%)  ^2^Design-based KruskalWallis test; Pearson's X^2: Rao & Scott adjustment | | | | |

| **Supplementary table 5:** Survey-weighted association Between Abdominal Aortic Calcification Score and outcomes in cancer types with representation across all three AAC strata* | | | | | | |
| --- | --- | --- | --- | --- | --- | --- |
|  | All-Cause Mortality | | | Cardiovascular Mortality | | |
| **AAC-24 Score** | **HR**^1^ | **95% CI**^1^ | **p-value** | **sHR**^1^ | **95% CI**^1^ | **p-value** |
| Per 1 unit increase in Score | 1.05 | 1.00, 1.10 | 0.04 | 1.08 | 1.01, 1.14 | 0.02 |
| By severity group |  |  |  |  |  |  |
| None (AAC24=0) | Reference | - | - | Reference | - | - |
| Moderate (AAC24= 1-6) | 0.98 | 0.54, 1.76 | 0.54 | 2.07 | 0.87, 4.95 | 0.09 |
| Severe (AAC24 >6) | 1.89 | 1.04, 3.44 | 0.04 | 2.81 | 1.17, 6.76 | 0.02 |
| ^1^HR = Hazard Ratio, sHR = Subdistribution Hazard Ratio, CI = Confidence Interval. Models were adjusted for sex, age, education, income, Hypertension, Diabetes Mellitus, Hyperlipidaemia, Smoking, BMI, Alcohol intake, Marital status, HDL, TG, Cancer site, Time from cancer diagnosis, Baseline CVD, and General Health perception. *Analysis excluded the following cancer sites: testis, stomach, pancreas, mouth/tongue/lip, liver, larynx, esophagus, brain, and bladder. | | | | | | |

| **Supplementary table 6:** Survey-weighted characteristics of participants without CVD and hypercalcemia at baseline (defined by adjusted calcium levels > 2.6 mmol/L) by Abdominal Aortic Calcification categories | | | | | |  |  |
| --- | --- | --- | --- | --- | --- | --- | --- |
| **Characteristic** | **Overall**  N = 17,492,167^1^ | **None (AAC24=0)**  N = 10,061,552^1^ | **Moderate (AAC24= 0-6)**  N = 4,323,770^1^ | **Severe**  **(AAC24 >6)**  N = 3,106,845^1^ | **p-value**^2^ | |  |
| **Unweighted records** | 352 (100%) | 182 (51.7%) | 103 (29.2%) | 67 (19%) |  | |  |
| **All-cause mortality** | 2,047,060 (12%) | 811,235 (8.1%) | 573,147 (13%) | 662,678 (21%) | 0.072 | |  |
| **CV Mortality** | 310,523 (1.8%) | 37,795 (0.4%) | 71,123 (1.6%) | 201,605 (6.5%) | 0.012 | |  |
| **Age in years** | 65 (56, 73) | 61 (54, 69) | 67 (59, 77) | 73 (65, 78) | <0.001 | |  |
| **Age group** |  |  |  |  | 0.011 | |  |
| 40-60 | 5,978,510 (34%) | 4,365,466 (43%) | 1,178,887 (27%) | 434,157 (14%) |  | |  |
| >60 | 11,513,658 (66%) | 5,696,087 (57%) | 3,144,883 (73%) | 2,672,688 (86%) |  | |  |
| **Sex** |  |  |  |  | 0.7 | |  |
| Male | 7,934,138 (45%) | 4,644,906 (46%) | 1,760,812 (41%) | 1,528,420 (49%) |  | |  |
| Female | 9,558,030 (55%) | 5,416,646 (54%) | 2,562,958 (59%) | 1,578,425 (51%) |  | |  |
| **Race/Hispanic origin** |  |  |  |  | 0.7 | |  |
| Mexican American | 492,113 (2.8%) | 263,182 (2.6%) | 158,811 (3.7%) | 70,119 (2.3%) |  | |  |
| Other Hispanic | 368,274 (2.1%) | 228,932 (2.3%) | 139,342 (3.2%) | 0 (0%) |  | |  |
| Non-Hispanic White | 15,259,072 (87%) | 8,799,235 (87%) | 3,693,630 (85%) | 2,766,206 (89%) |  | |  |
| Non-Hispanic Black | 723,662 (4.1%) | 418,263 (4.2%) | 180,996 (4.2%) | 124,403 (4.0%) |  | |  |
| Non-Hispanic Asian | 284,267 (1.6%) | 166,743 (1.7%) | 71,271 (1.6%) | 46,253 (1.5%) |  | |  |
| Other Race, Including Multi-racial | 364,779 (2.1%) | 185,196 (1.8%) | 79,719 (1.8%) | 99,864 (3.2%) |  | |  |
| **Education level** |  |  |  |  | 0.018 | |  |
| Less than High School | 363,290 (2.1%) | 152,817 (1.5%) | 151,464 (3.5%) | 59,009 (1.9%) |  | |  |
| High school or equivalent | 3,887,322 (22%) | 1,719,019 (17%) | 1,411,677 (33%) | 756,626 (24%) |  | |  |
| More than High school | 13,241,555 (76%) | 8,189,716 (81%) | 2,760,630 (64%) | 2,291,210 (74%) |  | |  |
| **Ratio of family income to poverty** |  |  |  |  | 0.6 | |  |
| <1.31 | 2,194,569 (13%) | 1,365,445 (14%) | 565,270 (13%) | 263,854 (8.5%) |  | |  |
| 1.31-1.85 | 1,463,350 (8.4%) | 691,690 (6.9%) | 389,151 (9.0%) | 382,509 (12%) |  | |  |
| 1.86-3.5 | 13,834,249 (79%) | 8,004,417 (80%) | 3,369,349 (78%) | 2,460,482 (79%) |  | |  |
| **Marital status** |  |  |  |  | 0.14 | |  |
| Married | 11,289,407 (65%) | 6,553,316 (65%) | 3,098,252 (72%) | 1,637,839 (53%) |  | |  |
| Widowed | 2,302,561 (13%) | 1,032,665 (10%) | 528,184 (12%) | 741,712 (24%) |  | |  |
| Divorced | 2,372,628 (14%) | 1,482,956 (15%) | 437,522 (10%) | 452,151 (15%) |  | |  |
| Separated | 190,770 (1.1%) | 43,785 (0.4%) | 126,737 (2.9%) | 20,248 (0.7%) |  | |  |
| Never Married | 1,089,711 (6.2%) | 819,471 (8.1%) | 64,250 (1.5%) | 205,991 (6.6%) |  | |  |
| Living with partner | 219,261 (1.3%) | 129,360 (1.3%) | 40,997 (0.9%) | 48,904 (1.6%) |  | |  |
| **Body Mass Index(kg/m**2)** | 28 (24, 32) | 28 (24, 33) | 28 (25, 31) | 28 (24, 32) | 0.8 | |  |
| **Systolic BP (mmHg)** | 127 (116, 140) | 125 (115, 137) | 127 (117, 140) | 131 (117, 147) | 0.6 | |  |
| **Diastolic BP (mmHg)** | 69 (61, 75) | 70 (62, 77) | 68 (62, 73) | 65 (59, 73) | 0.050 | |  |
| **Total Cholesterol(mmol/L)** | 4.94 (4.22, 5.72) | 5.04 (4.29, 5.84) | 4.86 (4.09, 5.40) | 4.68 (3.96, 5.84) | 0.089 | |  |
| **Direct HDL-Cholesterol (mmol/L)** | 1.29 (1.06, 1.68) | 1.32 (1.01, 1.68) | 1.22 (1.03, 1.53) | 1.40 (1.14, 1.73) | 0.3 | |  |
| **LDL-cholesterol (mmol/L)** | 2.93 (2.46, 3.39) | 2.97 (2.56, 3.45) | 2.83 (2.29, 3.33) | 2.72 (2.21, 3.38) | 0.12 | |  |
| **Triglyceride (mmol/L)** | 1.38 (1.04, 1.82) | 1.35 (0.96, 1.85) | 1.50 (1.13, 1.83) | 1.37 (1.05, 1.71) | 0.7 | |  |
| **Glycohemoglobin (%)** | 5.60 (5.30, 6.00) | 5.50 (5.30, 5.90) | 5.80 (5.50, 6.10) | 5.80 (5.50, 6.40) | 0.015 | |  |
| **Creatinine (umol/L)** | 80 (67, 94) | 80 (66, 91) | 83 (70, 103) | 80 (67, 94) | 0.2 | |  |
| **eGFR (mL/min/1.73 m²)** | 75 (63, 85) | 77 (65, 86) | 70 (56, 78) | 75 (59, 90) | 0.003 | |  |
| **Albumin (g/dL)** | 4.30 (4.10, 4.40) | 4.30 (4.10, 4.40) | 4.30 (4.10, 4.50) | 4.10 (4.00, 4.30) | 0.2 | |  |
| **Total calcium (mmol/L)** | 2.35 (2.30, 2.43) | 2.35 (2.30, 2.43) | 2.35 (2.30, 2.45) | 2.35 (2.30, 2.40) | 0.5 | |  |
| **Adjusted calcium (mmol/L)** | 2.17 (2.03, 2.30) | 2.16 (2.01, 2.27) | 2.16 (2.00, 2.30) | 2.25 (2.09, 2.37) | 0.2 | |  |
| **General health** |  |  |  |  | 0.4 | |  |
| Excellent | 1,996,881 (11%) | 1,164,538 (12%) | 542,094 (13%) | 290,249 (9.3%) |  | |  |
| Very good | 5,427,338 (31%) | 3,437,235 (34%) | 1,155,773 (27%) | 834,330 (27%) |  | |  |
| Good | 6,686,023 (38%) | 3,770,199 (37%) | 1,890,964 (44%) | 1,024,860 (33%) |  | |  |
| Fair | 2,448,137 (14%) | 1,104,736 (11%) | 643,178 (15%) | 700,223 (23%) |  | |  |
| Poor | 933,790 (5.3%) | 584,845 (5.8%) | 91,762 (2.1%) | 257,182 (8.3%) |  | |  |
| **Alcohol Consumption** |  |  |  |  | 0.3 | |  |
| None | 6,735,992 (39%) | 3,884,129 (39%) | 1,735,905 (40%) | 1,115,959 (36%) |  | |  |
| Moderate | 7,884,455 (45%) | 5,017,668 (50%) | 1,534,877 (35%) | 1,331,909 (43%) |  | |  |
| Heavy | 2,871,721 (16%) | 1,159,755 (12%) | 1,052,988 (24%) | 658,977 (21%) |  | |  |
| **Smoking** |  |  |  |  | 0.7 | |  |
| Never | 8,296,674 (47%) | 5,060,264 (50%) | 1,850,565 (43%) | 1,385,844 (45%) |  | |  |
| Former | 2,677,984 (15%) | 1,573,364 (16%) | 723,631 (17%) | 380,989 (12%) |  | |  |
| Current | 6,517,509 (37%) | 3,427,924 (34%) | 1,749,574 (40%) | 1,340,011 (43%) |  | |  |
| **Hypertension** | 9,845,803 (56%) | 4,925,074 (49%) | 3,072,620 (71%) | 1,848,110 (59%) | 0.038 | |  |
| **Hyperlipidemia** | 9,825,678 (56%) | 5,033,145 (50%) | 2,482,504 (57%) | 2,310,030 (74%) | 0.023 | |  |
| **Diabetes Mellitus** | 2,442,748 (14%) | 1,117,225 (11%) | 567,574 (13%) | 757,949 (24%) | 0.12 | |  |
| **Time from cancer diagnosis (years) (years)** | 26 (20, 30) | 22 (19, 28) | 26 (22, 32) | 31 (26, 33) | <0.001 | |  |
| **Number of Cancers** |  |  |  |  | 0.4 | |  |
| 1 | 15,759,562 (90%) | 9,313,021 (93%) | 3,824,225 (88%) | 2,622,316 (84%) |  | |  |
| 2 | 1,688,211 (9.7%) | 748,531 (7.4%) | 455,151 (11%) | 484,529 (16%) |  | |  |
| 3 | 44,394 (0.3%) | 0 (0%) | 44,394 (1.0%) | 0 (0%) |  | |  |
| ^1^n (%); Median (Q1, Q3). ^2^Pearson's X^2: Rao & Scott adjustment; Design-based KruskalWallis test | | | | | | | |

**Supplementary figure 2:** Survey-weighted Heatmap of Abdominal Aortic Calcification Distribution by Cancer Site in participants without CVD and hypercalcemia at baseline (defined by adjusted calcium levels > 2.6 mmol/L)

**
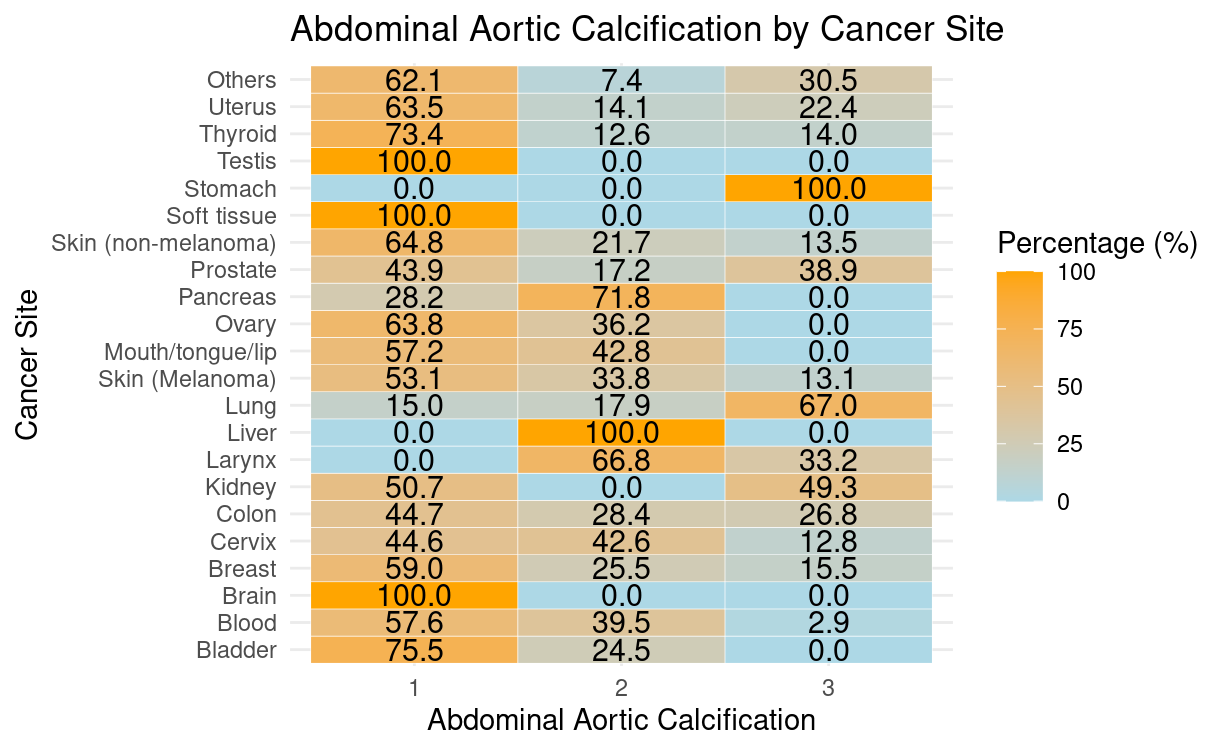
**

**Supplementary figure 3:** Survey-weighted Kaplan–Meier (KM) survival curves by Abdominal Aortic Calcification Severity in participants without CVD and hypercalcemia at baseline (adjusted calcium levels > 2.6 mmol/L)

for a) all-cause mortality, and b) CV mortality. P-value for log-rank (< 0.05).

**
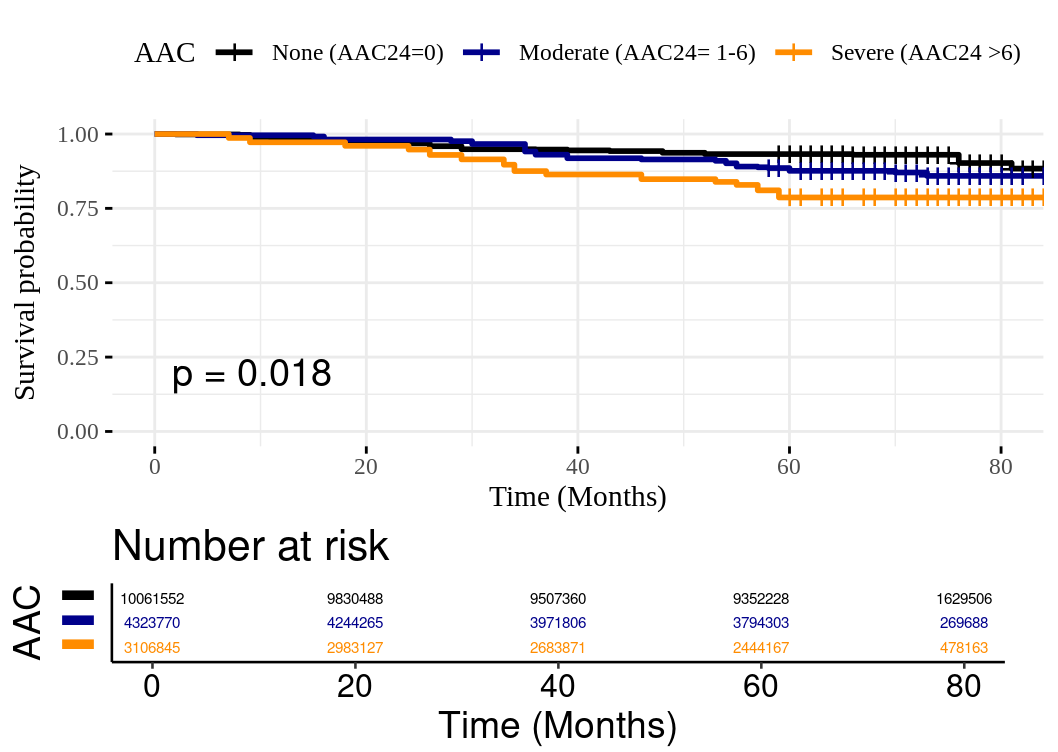
**

**
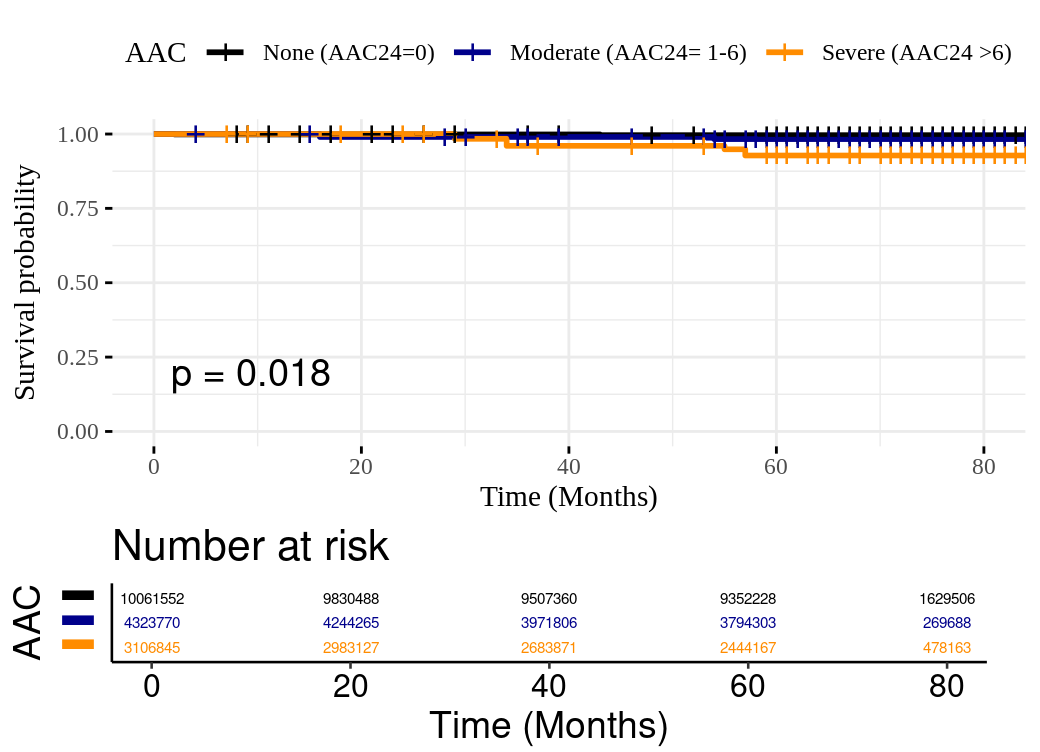
**

| **Supplementary table 7:** Survey-weighted association Between Abdominal Aortic Calcification Score and outcomes after excluding participants with baseline CVD and hypercalcemia (defined by adjusted calcium levels > 2.6 mmol/L) | | | | | | |
| --- | --- | --- | --- | --- | --- | --- |
| AAC-24 Score (continuous variable) | All-Cause Mortality | | | Cardiovascular Mortality | | |
| **Per 1 unit increase in Score** | **HR**^1^ | **95% CI**^1^ | **p-value** | **sHR**^1^ | **95% CI**^1^ | **p-value** |
| Model 1 (unadjusted) | 1.07 | 1.01, 1.14 | 0.009 | 1.12 | 1.02, 1.24 | 0.01 |
| Model 2^+^ (partially adjusted) | 1.06 | 1.01, 1.12 | 0.01 | 1.10 | 1.02, 1.19 | 0.007 |
| Model 3^++^ (fully adjusted) | 1.05 | 1.00, 1.11 | 0.042 | 1.07 | 1.00, 1.14 | 0.045 |
| **Model 4* (by severity group)** |  |  |  |  |  |  |
| None (AAC24=0) | Reference | - | - | Reference | - | - |
| Moderate (AAC24= 1-6) | 1.68 | 0.78, 3.60 | 0.17 | 1.13 | 0.86, 1.49 | 0.37 |
| Severe (AAC24 >6) | 2.73 | 1.22, 6.10 | 0.01 | 1.49 | 1.03, 2.15 | 0.03 |
| ^1^HR = Hazard Ratio, sHR = Subdistribution Hazard Ratio, CI = Confidence Interval. Model 1: unadjusted. ^+^Model 2: adjusted for sex, age, education, income, Hypertension, Diabetes Mellitus, Hyperlipidaemia, Smoking, BMI. ^++^ Mode 3: included adjustments for model 2 variables plus Alcohol intake, Marital status, HDL, TG, Cancer site, Time from cancer diagnosis , General Health perception. * Adjusted for all variables included in Model 3. | | | | | | |

| **Supplementary table 8:** Predictive performance of the fitted models in sensitivity analysis | | | | | |
| --- | --- | --- | --- | --- | --- |
| **All-cause mortality** | **Discrimination (C-statistic)** | | **Calibration (C-slope)** | | |
| Per 1 unit increase in Score (Model 1-3) | Original | Optimism-adjusted | | Original | Optimism- adjusted |
| Model 1 (unadjusted) | 0.60 | 0.60 | | 1 | 1.49 |
| Model 2^+^ (partially adjusted) | 0.73 | 0.67 | | 1 | 0.75 |
| Model 3^++^ (fully adjusted) | 0.73 | 0.66 | | 1 | 0.63 |
| Model 4* (by severity group) | 0.74 | 0.66 | | 1 | 0.63 |
| **CV mortality** |  |  | |  |  |
| Model 1 (unadjusted) | 0.54 | 0.53 | | 1 | 2.06 |
| Model 2^+^ (partially adjusted) | 0.63 | 0.61 | | 1 | 0.84 |
| Model 3^++^ (fully adjusted) | 0.64 | 0.62 | | 1 | 0.75 |
| Model 4* (by severity group) | 0.64 | 0.61 | | 1 | 0.74 |
| Model 1: unadjusted. ^+^Model 2: adjusted for sex, age, education, income, Hypertension, Diabetes Mellitus, Hyperlipidaemia, Smoking, BMI. ^++^ Model 3: included adjustments for model 2 variables plus Alcohol intake, Marital status, HDL, TG, Cancer site, Time from cancer diagnosis, Baseline CVD, General Health perception. * Adjusted for all variables included in Model 3. | | | | | |
